# Therapeutic Targeting Potential of Novel Silver Nanoparticles Coated with Anti-CD20 Antibody against Chronic Lymphocytic Leukemia

**DOI:** 10.3390/cancers15143618

**Published:** 2023-07-14

**Authors:** Francesco Maria Adamo, Estevao Carlos Silva Barcelos, Filomena De Falco, Erica Dorillo, Chiara Rompietti, Daniele Sorcini, Arianna Stella, Beatrice Del Papa, Stefano Baldoni, Angela Esposito, Clelia Geraci, Roberta Arcaleni, Chiara Pennetta, Francesco Ragonese, Lorenzo Moretti, Mariagrazia Mameli, Mauro Di Ianni, Emanuela Rosati, Bernard Fioretti, Paolo Sportoletti

**Affiliations:** 1 Department of Medicine and Surgery, Institute of Hematology and Center for Hemato-Oncology Research (CREO), University of Perugia, Santa Maria della Misericordia Hospital, 06129 Perugia, Italy; francesco91adamo@gmail.com (F.M.A.); estevaocarlosbarcelos@gmail.com (E.C.S.B.); filomena.defalco@unipg.it (F.D.F.); erica.do@hotmail.it (E.D.); rompiettic@yahoo.it (C.R.); daniele.sorcini@unipg.it (D.S.); arianna.stella89@gmail.com (A.S.); beadel@libero.it (B.D.P.); stefobaldo@gmail.com (S.B.); angela.esposito13@gmail.com (A.E.); cleliagerac@gmail.com (C.G.); arcaleniroberta@gmail.com (R.A.); lorenzo.moretti@ospedale.perugia.it (L.M.); mgrazia.mameli@ospedale.perugia.it (M.M.); 2 Postgraduate Program in Biotechnology, Federal University of Espírito Santo, Vitória 29043-900, Brazil; 3 Department of Medicine and Sciences of Aging, “G. d’Annunzio” University of Chieti-Pescara, 66100 Chieti, Italy; mauro.diianni@unich.it; 4 Department of Chemistry, Biology and Biotechnologies, University of Perugia, 06123 Perugia, Italy; pennetta90@gmail.com (C.P.); francescoragonese85@gmail.com (F.R.); 5 Department of Medicine and Surgery, Biosciences and Medical Embryology Section, University of Perugia, 06129 Perugia, Italy; emanuela.rosati@unipg.it

**Keywords:** chronic lymphocytic leukemia, drug mechanisms, preclinical models

## Abstract

**Simple Summary:**

Chronic lymphocytic leukemia (CLL) is an incurable hematological disorder, representing an unmet need within the field of cancer therapy. In this study, we highlighted the cytotoxicity of silver nanoparticles (AgNPs) against CLL cells and demonstrated the synergistic activity of AgNPs with drugs currently used in CLL, such as Venetoclax and Ibrutinib, which can be exploited for potential combined therapies. Furthermore, the conjugation of AgNPs with the anti-CD20 antibody Rituximab (AgNPs@Rituximab) increased the specificity and cytotoxicity of AgNPs toward CLL cells in vitro. AgNPs@Rituximab also extended the survival of CLL xenograft models compared to each unconjugated single agent. These data provided evidence that AgNPs@Rituximab could also overcome the non-specific distribution of AgNPs in vivo, thus increasing the selective elimination of CLL cells. The findings that emerged from this study could provide the rationale for further investigations aimed at defining a potential clinical application of nanotechnologies in the context of CLL therapy.

**Abstract:**

Background: Chronic lymphocytic leukemia (CLL) is an incurable disorder associated with alterations in several pathways essential for survival and proliferation. Despite the advances made in CLL therapy with the new target agents, in some cases, relapses and resistance could occur, making the discovery of new alternatives to manage CLL refractoriness necessary. To provide new therapeutic strategies for CLL, we investigated the anti-leukemic activity of silver nanoparticles (AgNPs), whose impact on CLL cells has been poorly explored. Methods: We studied the action mechanisms of AgNPs in vitro through flow cytometry and molecular analyses. To improve the bioavailability of AgNPs, we generated AgNPs coated with the anti-CD20 antibody Rituximab (AgNPs@Rituximab) and carried out imaging-based approaches and in vivo experiments to evaluate specificity, drug uptake, and efficacy. Results: AgNPs reduced the viability of primary CLL cells and the HG-3 cell line by inducing an intrinsic apoptotic pathway characterized by Bax/Bcl-2 imbalance, caspase activation, and PARP degradation. Early apoptotic events triggered by AgNPs included enhanced Ca^2+^ influx and ROS overproduction. AgNPs synergistically potentiated the cytotoxicity of Venetoclax, Ibrutinib, and Bepridil. In vitro, the AgNPs@Rituximab conjugates were rapidly internalized within CLL cells and strongly prolonged the survival of CLL xenograft models compared to each unconjugated single agent. Conclusions: AgNPs showed strong anti-leukemic activity in CLL, with the potential for clinical translation in combination with agents used in CLL. The increased specificity of AgNPs@Rituximab toward CLL cells could be relevant for overcoming in vivo AgNPs’ non-specific distribution and increasing their efficacy.

## 1. Introduction

In chronic lymphocytic leukemia (CLL), advances in gene expression profiling have revealed the existence of an extreme molecular heterogeneity that drives aggressiveness and responses to therapy [1,2]. In the last few decades, new therapeutic alternatives have been developed with the aim of improving CLL treatment. Some of these compounds act on several pathways aberrantly activated or expressed in CLL and include the inhibitor of Bruton’s tyrosine kinase (BTK) Ibrutinib and the inhibitor of B-cell lymphoma 2 protein (Bcl-2) Venetoclax [3]. Although the majority of patient responses to these agents have been long lasting, relapses occur, especially in the high-risk patient population, making it necessary to investigate new therapeutic strategies [4].

Recently, nanotechnologies have attracted the interest of researchers for their versatile and functional properties that can further improve cancer treatment [5]. The stability conferred by materials of synthesis and the small sizes of nanoparticles (NPs) enhance their ability to overcome biological barriers and facilitate their intracellular uptake, improving the cytotoxic effect of therapy. In addition, their high surface-to-volume ratio allows for functionalizing NPs’ superficies with specific ligands and offers the possibility of loading multiple molecules to deliver into cancer cells, conferring tumor-targeted therapy properties [6]. Recent preclinical evidence has shown the potential use of silver NPs (AgNPs) in several neoplasms including cervical cancer, hepatocellular carcinoma [7], and hematological malignancies such as chronic myeloid leukemia [8,9], lymphoma [10], and acute myeloid leukemia [11]. However, their effects on CLL cells and the possibility that AgNPs can be utilized for the development of novel nanotherapeutic molecules specifically targeting CLL cells remain to be investigated. 

Here, we showed that AgNPs can lead CLL cells to cell death through the activation of the mitochondrial apoptosis machinery, triggered by changes in intracellular calcium (Ca^2+^) concentration and the subsequent accumulation of reactive oxygen species (ROS). In addition, we demonstrated that AgNPs synergized with new target agents used in CLL therapy, and displayed high efficacy and specificity against CLL when they were conjugated with an anti-CD20 antibody.

## 2. Methods

### 2.1. Synthesis of Silver Nanoparticles and Conjugations with Rituximab

For the synthesis of silver nanoparticles (AgNPs), a solution was obtained by mixing 2.5 mM AgNO_3_ and 2.5 mM citric acid trisodium salt (AgNPs@cit). An aqueous solution of NaBH_4_ was added dropwise to the mixture at a final concentration of 3.6 mM under vigorous stirring. The solution was stirred in the dark at 4 °C for 30 min. For the conjugation of AgNPs with Rituximab or with IgG1 used as an isotype control, first, an aqueous solution of Poly (ethylene glycol) 2-mercaptoethyl ether acetic acid average (SH-PEG-COOH, 1 KDa, 1 mg/mL) was added to a suspension of AgNPs@cit at a *v*/*v* ratio of 1:19 (AgNPs@PEG). The mixture was stirred for 30 min. Nanoparticles were centrifuged for 40 min at 20,000× *g*. The supernatant was discarded, and the pellet was suspended in dH_2_O. Then, AgNPs@PEG (100 nM) was mixed at a volume ratio of 20:1, with a solution obtained by mixing 1-Ethyl-3-(3-dimethylaminopropyl) carbodiimide (EDC, 60 mg/mL) and N-Hydroxysulfosuccinimide (S-NHS, 30 mg/mL) at a volume ratio of 1:1, under stirring for 15 min. Finally, AgNPs conjugated with Rituximab (AgNPs@Rituximab) or IgG1 (AgNPs@IgG1) were obtained by mixing the previously obtained solution with Rituximab (100 mg/10 mL, Rixathon^®^, Sandoz s.p.a., Milan, Italy) or IgG1 (100 mg/10 mL) at a volume ratio of 1:1 under soft stirring for 1 h. The unreacted Rituximab or IgG1 was removed through centrifugation for 30 min at 10,000× *g*. The supernatants were discarded, and the amounts of antibodies conjugated with AgNPs were considered similar to those reported with comparable procedures, which were estimated at 5 ± 1 Abs/AgNPs [12]. The concentration of each variant of AgNPs (AgNPs@cit, AgNPs@PEG, AgNPs@Rituximab, and AgNPs@IgG1) expressed in nM was estimated according to Paramelle et al. [13], assuming that they had similar molar extinction coefficients (ε). Explicative UV–Vis spectra of each variant of AgNPs recorded during the synthesis of AgNPs@Rituximab are reported in Figure S1, together with TEM imaging analyses of AgNPs@Rituximab, which were performed to better characterize the nanomaterials.

### 2.2. Patients 

This study was approved by the Ethics Committee of Perugia University, and all patients (*n* = 23) signed informed consent in accordance with the Declaration of Helsinki. Table S1 summarizes the clinical and biological characteristics of CLL patients.

### 2.3. Isolation of Primary CLL Cells 

Primary CLL cells were isolated from patient peripheral blood samples through Ficoll density gradient centrifugation, followed by sheep erythrocyte rosetting. The average purity of CD19^+^/CD5^+^ CLL cells was 94.59 ± 3.09%, as determined using flow cytometry (EPICS-XLMCL; Beckman Coulter, Fullerton, CA, USA) analysis performed using anti CD45, CD19, CD5 (mAb**s**) on 7AAD negative cells (all from Beckman Coulter). 

### 2.4. In Vitro Treatment 

Primary CLL cells were cultured for 24 h at 37 °C in a 5% CO_2_ atmosphere in complete medium, consisting of RPMI 1640 supplemented with 10% heat-inactivated fetal bovine serum (Gibco, Gaithersburg, MD, USA), 2 mM L-glutamine, and 100 U/mL penicillin, 100 μg/mL streptomycin (all from Invitrogen, Milan, Italy), and containing 1 nM AgNPs, or citrate as control. HG-3 CLL cells and the acute myeloid leukemia (AML) OCI-AML 3 cell line (both obtained from ATCC, Manassas, VA, USA) were cultured for 24 h at 37 °C in a 5% CO_2_ atmosphere in complete medium containing 1 nM AgNPs, 10 μg/mL Rituximab, 1 nM AgNPs@Rituximab, 1 nM AgNPs@IgG1, or citrate as control. In pharmacological studies, HG-3 cells were treated with 2 nM Venetoclax (LC Laboratories, Woburn, MA, USA), 10 μM Ibrutinib (Selleck Chemicals, Houston, TX, USA), and 2.5 µM Bepridil (Sigma Aldrich, Saint Louis, MO, USA), or DMSO as control for 24 h.

### 2.5. Flow Cytometry Analyses

Cell viability/apoptosis was assessed using Annexin V-FITC/Propidium Iodide (An V/PI) staining (Immunotech, Beckman Coulter, Fullerton, CA, USA). The measurement of Ca^2+^ flux was performed using the Fluo-4 Direct^TM^ calcium Assay Kit (Life technologies, Thermo Scientific, Eugene, OR, USA) following the manufacturer’s instructions.

Intracellular ROS levels were evaluated using ROS-sensitive probe 2′,7′-dichlorodihydrofluorescein diacetate (H2-DCFDA, Invitrogen, Milan, Italy). H2-DCFDA was dissolved in DMSO to obtain a 10 mM stock solution and further diluted at 5 µM before use as a staining solution. HG-3 cells were incubated with 5 µM staining solution in PBS in the dark for 30 min at 37 °C before treatment with 1 nM AgNPs for different timepoints (from 1 to 6 h). After washing and resuspension in PBS, cells were immediately analyzed with flow cytometry (FACS Canto, BD Biosciences, San Jose, CA, USA). Changes in mitochondrial membrane potential were analyzed using the Mitotracker Red CMXRos (Invitrogen Carlsbad, CA, USA). After treatment, collected cells were washed and incubated with the Mitotracker Red probe at a final concentration of 100 nM. After incubation for 20 min at room temperature in the dark, cells were centrifuged, resuspended, and analyzed with flow cytometry (FACS Canto, BD Biosciences, San Jose, CA, USA). Data analyses were performed with FlowJo software version 10 (FlowJo, LLC, Ashland, OR, USA). 

### 2.6. Cell Viability Assay

After treatments, 100 µL of cell suspension was transferred in an opaque 96-well plate and 20 µL of CellTiter-Glo^®^ Luminescent Cell Viability Assay (Promega, Madison, WI, USA) was added to each well. After 2 min of vigorous shaking and 10 min standing at room temperature, luminescence was measured with a Spark^®^ Multimode Microplate Reader (Tecan, Männedorf, Switzerland) over 500 ms of integration time. Data were expressed normalized to vehicle. 

### 2.7. Western Blot

Whole-cell lysates were obtained using the RIPA lysis buffer containing a protease/phosphatase inhibitor cocktail (Sigma-Aldrich, St. Louis, MO, USA). Protein concentration was determined via Bradford assay, and Western blot was performed as previously described [14] using the primary antibodies listed in Table S2. Horseradish peroxidase-conjugated secondary antibodies (Cell signaling Technology, Beverly, MA, USA) together with Supersignal chemiluminescent substrate (Life Technologies, Thermo Scientific, Eugene, OR, USA) were used to detect a signal on a ChemiDoc^TM^ MP Imaging System (Bio-Rad, Milan, Italy). Densitometric analysis was performed using ImageLab software 6.1 (Bio-Rad, Milan, Italy).

### 2.8. Real-Time qPCR

RNA was extracted using the RNeasy Plus Kits (Qiagen, Hilden, Germany) and cDNA was obtained using the Prime Script RT Master Mix (Takara Bio, Kusatsu, Japan). We used the PCR Master Mix Power SYBER Green and the 7300 HT Real-Time PCR System (Applied Biosystems, Waltham, MA, USA). The primer sequences are included in Table S3. Relative fold change was normalized to GAPDH and calculated using the 2^−ΔΔCt^ method.

### 2.9. Ca^2+^ Imaging

HG-3 cells were incubated with the FURA-2-AM (3 μM; Sigma-Aldrich, Burlington, MA, USA) for 45 min and extensively washed with external solution of the following composition (in mM): NaCl 140, KCl 5, CaCl_2_ 2, MgCl_2_ 2, MOPS 5, and glucose 10, at pH 7.4. Cells were continuously perfused using a gravity-driven perfusion system, focally oriented onto the field of interest. The estimation of the intracellular free Ca^2+^ concentration was reported as a change in the ratio between fluorescence emission at 510 nm obtained with 340 and 380 nm excitation wavelengths (optical filters and dichroic beam splitter were from Lambda DG4, Shutter Instruments, Novato, CA, USA) after AgNPs or citrate exposure. Ratiometric data were acquired every 3 s and fluorescence determinations were performed using the Zeiss fluorescence microscopy system (Axiozoom V16 and Axiocam 502 mono). The acquisition and analysis were driven using the ZEN 2 software 1.0 (Zeiss, Jena, Germany).

### 2.10. Transmission Electron Microscopy (TEM) Analysis

Samples were preserved in a 2.5% glutaraldehyde/0.1 M sodium cacodylate buffer overnight at 4 °C. Samples were then treated with 1% OsO_4_/0.1 M sodium cacodylate buffer and dehydrated using ethanol solutions of increasing concentrations before embedding in the EPON epoxy resins. Ultrathin sections were obtained and treated with 1% uranyl acetate and 1% lead citrate. Samples were analyzed with TEM (University Center for the Electron Microscopy, University of Perugia) using a Philips EM 400 electron microscope (HT range 20–120 kV). 

### 2.11. Xenograft Model 

A total of 1 × 10^6^ HG-3 cells were intravenously (i.v.) injected into 8–15-week-old non-irradiated NSG mice. After 3 days, mice were randomly divided into four groups (*n* = 6/group): Citrate, AgNPs, Rituximab, and AgNP@Rituximab groups. Drugs were administered by intraperitoneal injection 4 times every 4 days at about 10 mg/kg concentration. The four groups of mice were then monitored daily, and the survival was estimated according to the Kaplan–Meier method. Animal studies were conducted in accordance with the European guidelines and received the approval of the Italian Ministry of Health (authorization #971/2020-PR) and of the Animal Care and Use Committee of our institution. 

### 2.12. Statistical Analysis

Statistical analyses were performed with the GraphPad Software version 8 (GraphPad Software Inc., La Jolla, CA, USA). Data were presented as mean ± SD and statistical differences between mean values were evaluated using nonparametric tests such as Wilcoxon for paired data and Mann–Whitney for unpaired data. In animal studies, survival was estimated from the start of treatment to death using the Kaplan–Meier method, and the log-rank test was used to compare differences between survival curves. For all analyses, *p* < 0.05 was considered statistically significant. 

## 3. Results

### 3.1. AgNPs Reduced Viability of Primary CLL Cells

**Figure 1:**
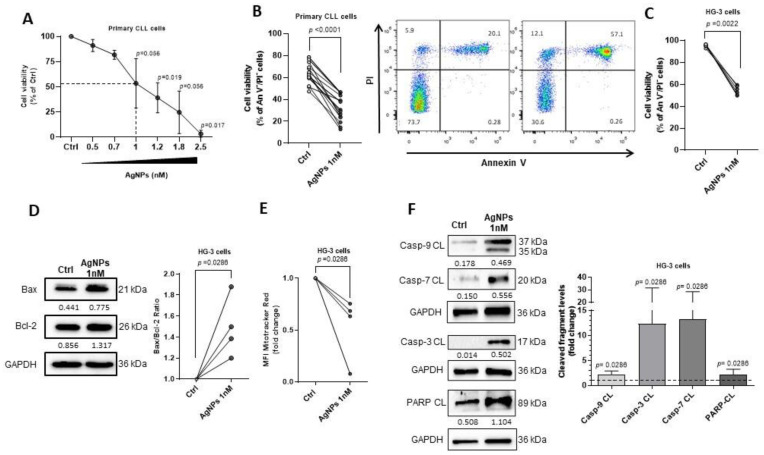
AgNPs reduce viability of CLL cells by inducing intrinsic apoptotic pathway. (**A**–**F**) CLL cells were cultured for 24 h with the indicated concentrations of AgNPs or citrate as vehicle control (Ctrl). (**A**–**C**) Cell viability was determined using An V/PI flow cytometry assay. (**A**) Dose-response curve of CLL cells from 6 patients to AgNPs. Data are presented as the mean ± SD of the percentage of viable An V^−^/PI^−^ cells normalized to Ctrl set as 100%. Dotted line represents EC50 value. (**B**) Left, dot and line graph of viable An V^−^/PI^−^ CLL cells from 18 patients. *p* values were calculated according to Wilcoxon’s paired test. Right, representative dot plots showing the percentage of viable An V^−^/PI^−^, early apoptotic An V^+^/PI^−^, late apoptotic An V^+^/PI^+^, and necrotic An V^−^/PI^+^ CLL cells. (**C**) Dot and line graph of viable An V^−^/PI^−^ HG-3 cells from six independent experiments. *p* values were calculated according to Mann–Whitney unpaired test. (**D**) Left, representative Western blot of Bax and Bcl-2. The number under each lane indicates the densitometry value of the band relative to GAPDH levels. Right, dot and line graph of Bax/Bcl-2 ratio from four independent experiments. Data are normalized to Ctrl set as 1. *p* values were calculated according to Mann–Whitney unpaired test. (**E**) Dot and line graph of mean fluorescence intensity (MFI) of mitochondrial membrane potential from four independent experiments. Data are normalized to Ctrl set as 1. *p* values were calculated according to Mann–Whitney unpaired test. (**F**) Left, representative Western Blot of the cleaved caspase-9, -3, -7, and PARP fragments from four independent experiments. The number under each lane indicates the densitometry value of the band relative to GAPDH levels. Right, column bar graph of densitometry data of each cleaved fragment in AgNPs-treated cells, normalized to GAPDH levels, compared to Ctrl set as 1. Data are presented as the mean ± SD of four independent experiments. *p* values were calculated according to Mann–Whitney unpaired test. The uncropped blots are shown in the Figure S6.

In order to assess the sensitivity of CLL cells to AgNPs, primary cells (*n* = 6) were incubated with various doses of AgNPs ranging from 0.5 to 2.5 nM using citrate as the vehicle control. Cell viability was evaluated via Annexin V/PI assay after 24 h incubation. Results showed that AgNPs reduced CLL cell viability in a concentration-dependent manner, and the half-maximal response (EC50) was obtained with 1 nM concentration (Figure 1A). We then investigated the effect of 24 h treatment with 1 nM AgNPs on 18 CLL samples. The results showed that cell viability was significantly reduced to 33.34 ± 11.17% compared to 64.65 ± 9.2% of the control (Figure 1B). AgNPs significantly increased the percentage of An V^+^/PI^+^ late apoptotic cells as compared to control (52.54 ± 15.58% vs. 25.28 ± 8.79%), with only a marginal effect on An V^+^/PI^−^ early apoptotic cells (12.43 ± 8.68% vs. 11.58 ± 5.33%; Figure S2A). With the aim to exclude variables due to the heterogeneity of primary CLL cells, we evaluated the anti-leukemic effect of 1 nM AgNPs on the human HG-3 CLL cell line. In keeping with data obtained in primary cells, we found that AgNPs treatment induced a significant reduction in viable cells (54.42 ± 4.07% vs. 95.33 ± 1.41%; Figure 1C) and a significant increase in late apoptotic cells compared to the control (39.22 ± 3.48% vs. 2.22 ± 0.91%; Figure S2B).

### 3.2. AgNPs Induced Mitochondrial Apoptosis in HG-3 CLL Cells 

In order to gain insight into the mechanisms involved in the cytotoxic effects of AgNPs, we measured different markers of apoptosis in HG-3 cells upon treatment. We found an increased expression of the pro-apoptotic Bax protein and unchanged levels of the anti-apoptotic Bcl-2. This led to a significant imbalance in the Bax/Bcl-2 ratio (1.49 ± 0.29-fold increase vs. control set as 1; Figure 1D), indicating a susceptibility of CLL cells to an intrinsic apoptosis pathway. When we performed this same analysis using primary CLL cells, we found similar results to those obtained with HG-3 cells. Specifically, we observed a 2.44 ± 1.73-fold increase in the Bax/Bcl-2 ratio after AgNPs treatment compared to the control set as 1 (Figure S3).

We also observed that HG-3 cells treated with AgNPs showed a decreased mitochondrial membrane potential registered as mean fluorescence intensity (MFI) by flow cytometry (0.53 ± 0.31-fold decrease; Figure 1E), indicating that the increased Bax expression could result in the rupture of the mitochondrial outer membrane. It is known that mitochondrial outer membrane permeabilization (MOMP) triggers a cascade of downstream caspase activation involved in the apoptotic process induced by AgNPs [15,16]. Figure 1F shows that the AgNPs treatment significantly increased the levels of cleaved caspase-9 (2.23 ± 0.67-fold increase vs. control set as 1), caspase-3 (12.38 ± 19.19-fold increase vs. control set as 1), and caspase-7 (13.23 ± 15.29-fold increase vs. control set as 1) in HG-3 cells. Additionally, we detected increased levels of cleaved PARP fragment (2.14 ± 1.11-fold increase vs. control set as 1), a known target of caspase activation (Figure 1F). Altogether, these data demonstrated that AgNPs induced CLL cell injury through an intrinsic apoptotic mechanism. 

### 3.3. AgNPs Modulated the Expression of Calcium Channel in CLL Cells 

**Figure 2:**
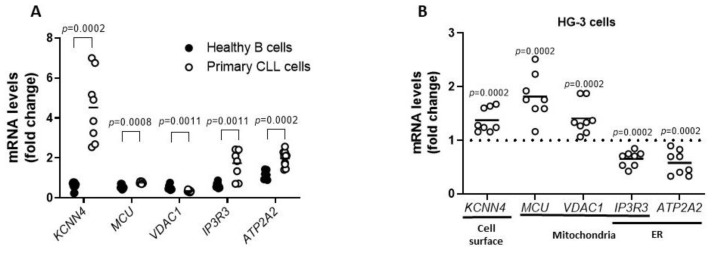
Altered expression of Ca^2+^ channel in CLL cells. (**A**) Scatter plot with data points shows the analysis of mRNA expression of the Ca^2+^ channels *KCNN4*, *MCU*, *VDAC1*, *IP3R3*, and *ATP2A2* in healthy B cells (*n* = 8) and CLL cells (*n* = 8). mRNA levels were normalized to GAPDH and represented as fold change compared to mRNA means of healthy B cells. *p* values were calculated according to Mann–Whitney unpaired test. (**B**) Scatter plot with data points shows the analysis of *KCNN4*, *MCU*, *VDAC1*, *IP3R3*, and *ATP2A2* gene expression in HG-3 cells treated for 6 h with 1 nM AgNPs or vehicle control (Ctrl) in eight independent experiments. mRNA levels were normalized to GAPDH and represented as fold change compared to Ctrl set as 1. *p* values were calculated according to Mann–Whitney unpaired test.

As several studies have reported that AgNPs modulate cellular Ca^2+^ homeostasis [7], we first characterized Ca^2+^ homeostasis in CLL cells as a potential target for nanoparticle effects. We compared the expression of the Ca^2+^ channel mRNA of primary CLL cells with that of healthy B cells (*n* = 8, for both). Specifically, we analyzed Ca^2+^ channels localized on the cell surface, on mitochondria and endoplasmic reticulum (ER) membranes, or associated with the ER-mitochondria crosstalk [17,18,19,20,21]. Figure 2A showed that CLL cells exhibited a significant up-regulation of *KCNN4* (4.52 ± 1.73 vs. 0.58 ± 0.2), *MCU* (0.76 ± 0.05 vs. 0.56 ± 0.11), *IP3R3* (1.74 ± 0.73 vs. 0.62 ± 0.13), and *ATP2A2* (2.12 ± 0.29 vs. 1.17 ± 0.23), and a down-regulation of *VDAC1* (0.35 ± 1.73 vs. 0.51 ± 0.12). These results suggested that alterations in these regulators of Ca^2+^ homeostasis represented a hallmark of CLL cells, which might render them sensitive to Ca^2+^-targeting agents. 

To further define Ca^2+^ homeostasis as a vulnerability targeted by AgNPs, we measured mRNA levels of Ca^2+^ modulators in HG-3 cells treated with AgNPs (1 nM for 6 h). As shown in Figure 2B, q-PCR analysis revealed that AgNPs significantly up-regulated the expression of *KCNN4*, *MCU*, and *VDAC1* (1.37 ± 0.22, 1.82 ± 0.41 and 1.41 ± 0.3, respectively), whereas *IP3R3* and *ATP2A2* levels were down-regulated, compared to the control set as 1 (0.65 ± 0.14 and 0.58 ± 0.23, respectively). These results indicated that AgNPs deregulated important channels involved in Ca^2+^ homeostasis in CLL cells. 

### 3.4. AgNPs Increased Ca^2+^ Influx and Stimulated ROS Production in HG-3 Cells 

**Figure 3:**
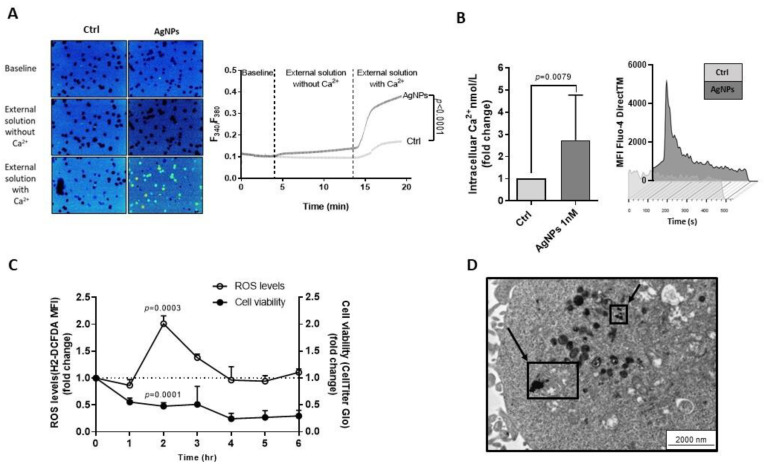
AgNPs increase Ca^2+^ influx and stimulate ROS production in HG-3 cells. (**A**) Fura-2 ratiometric images (4× magnification; **left**) and superimpose scatter plots (**right**) show changes in the cytosolic Ca^2+^ concentrations in HG-3 cells treated with 1 nM AgNPs or vehicle (Ctrl) in two different experimental conditions: external solution without or with Ca^2+^. *p* values were calculated according to Mann–Whitney unpaired test. (**B**) Left, column bar graph shows Ca^2+^ nmol/L increase in HG-3 in response to 1 nM AgNPs. Data are presented as mean ± SD of five independent experiments. Right, representative time-laps of Fluo-4 DirectTM changes after 1 nM AgNPs treatment, registered as mean fluorescence intensity (MFI). *p* values were calculated according to Mann–Whitney unpaired test. (**C**) Effects of 1 nM AgNPs on ROS generation, assessed by H2-DCFDA assay, and on cell viability assessed by CellTiter Glo assay at various time-points compared to Ctrl set as 1. Data are presented as the mean ± SD of three independent experiments. (**D**) TEM image of ultrathin sections of HG-3 cells treated with 1 nM AgNPs for 2 h shows particles internalized near to mitochondria, visible in the cells as black and electron-dense spots indicated by arrows. Scale bar represented 200 nm.

To better define whether AgNPs target Ca^2+^ homeostasis in CLL, we measured the intracellular Ca^2+^ concentration in AgNPs-treated HG-3 cells. As shown in Figure 3A, the FURA-2-AM Ca^2+^ imaging assay demonstrated a significant increase in intracellular Ca^2+^ concentration in AgNPs-treated cells compared to control (0.380 ± 0.058 vs. 0.11 ± 0.005). When replacing Ca^2+^ from the external solution with magnesium, intracellular Ca^2+^ levels were similar in AgNPs-treated cells compared to control (Figure 3A). These data suggest that Ca^2+^ ions incorporated by HG-3 cells after AgNPs exposure were derived from the extracellular environment. Similar results were obtained with the “Fluo-4 DirectTM Calcium Assay” performed using flow cytometry. In particular, the baseline MFI of cells labeled with the Fluo-4 was significantly increased after the addition of AgNPs compared to control (2.74 ± 2.03-fold increase vs. control set as 1) (Figure 3B).

Based on the evidence that high cytosolic Ca^2+^ levels stimulate mitochondria to produce high amounts of ROS [22], we investigated ROS levels in HG-3 cells treated with AgNPs at different time points ranging from 1 to 6 h. Results revealed the highest ROS production after 2 h treatment (1.77 ± 0.49-fold increase vs. control set as 1), considering the time at which cell viability was significantly reduced to 51.27 ± 0.08%, when compared to control (Figure 3C). These data indicated that AgNPs induced CLL cell cytotoxicity by increasing intracellular Ca^2+^ levels and ROS overproduction. 

We then performed Transmission Electron Microscopy (TEM) analysis in HG-3 cells, showing that AgNPs localized in close proximity to mitochondria after 2 h treatment (Figure 3D). These results confirmed the cellular uptake of AgNPs and suggested that AgNPs might directly interfere with mitochondrial functions through a physical interaction with mitochondria structures.

### 3.5. AgNPs Potentiated the Cytotoxic Activity of Agents Targeting Ca^2+^ Homeostasis and Mitochondria Functions in HG-3 Cells

We tested the combination of AgNPs and selected drugs known to target mitochondrial integrity and/or Ca^2+^ homeostasis on HG-3 cell viability.

**Figure 4:**
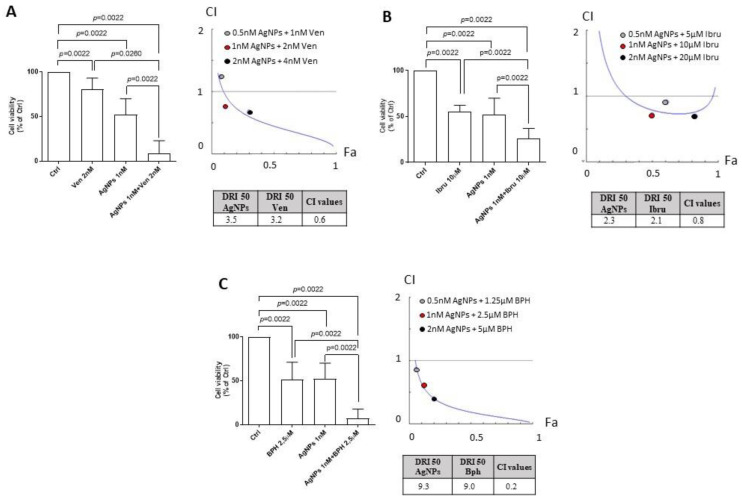
AgNPs potentiate the cytotoxicity of agents targeting Ca^2+^ homeostasis and mitochondria functions in HG-3 cells. (**A**) Left, column bar graph of HG-3 cell viability, evaluated by CellTiter Glo assay, after 24 h incubation with vehicle control (Ctrl) set as 100% or with 1 nM AgNPs and 2 nM Venetoclax alone or in combination. Data are presented as the mean ± SD of six independent experiments. Right (top), combination index plot (CI) of HG-3 cells treated with AgNPs and Venetoclax. Right (bottom), DRI 50 values. (**B**) Left, column bar graph of HG-3 cell viability, evaluated by CellTiter Glo assay, after 24 h incubation with vehicle control (Ctrl) set as 100% or with 1 nM AgNPs and 10 μM Ibrutinib alone or in combination. Data are presented as the mean ± SD of six independent experiments. Right (top), combination index plot (CI) of HG-3 cells treated with AgNPs and Ibrutinib. Right (bottom), DRI 50 values. (**C**) Left, column bar graph of HG-3 cell viability, evaluated with CellTiter Glo assay, after 24 h incubation with vehicle control (Ctrl) set as 100% or with 1 nM AgNPs and 2.5 μM Bepridil alone or in combination. Data are presented as the mean ± SD of six independent experiments. Right (top), combination index plot (CI) of HG-3 cells treated with AgNPs and Bepridil. Right (bottom), DRI 50 values. CI < 1 indicates synergism, CI = 1 indicates additive effect, CI > 1 indicates antagonism. *p* values were calculated according to Mann–Whitney unpaired test.

(i) We used the selective Bcl-2 inhibitor Venetoclax based on its role in inducing MOMP [23]. Figure 4A (left) showed that the combined treatment with AgNPs and Venetoclax potentiated the cytoxicity of each single agent, resulting in an increased reduction in cell viability compared to each single drug. Specifically, cell viability was significantly reduced to 70.08 ± 32.97% by Venetoclax, to 28.84 ± 19.79% by AgNPs, and to 8.98 ± 13.97% by a drug combination as compared to controls set to 100%. To determine whether the AgNPs/Venetoclax combination was synergic or additive, we conducted cytotoxicity tests at several drug concentrations and used the Chou–Talalay model. The combination index (CI) plot confirmed the synergistic effect of drug combination with 1 nM AgNPs + 2 nM Venetoclax and 2 nM AgNPs + 4 nM Venetoclax (Figure 4A, right top).
(ii) We tested the BTK inhibitor Ibrutinib and AgNPs combination, since Ibrutinib sensitizes cancer cells to ROS inductor agents [24]. As shown in Figure 4B (left), AgNPs potentiated the cytotoxic activity of Ibrutinib. Indeed, the viability of HG-3 cells was decreased to 57.77 ± 6% by Ibrutinib, to 63.87 ± 3.67% by AgNPs, and to 25.79 ± 11.3% by the drug combination as compared to controls set to 100%. The Chou–Talalay analysis revealed synergism between AgNPs and Ibrutinib (Figure 4B, right top).
(iii) We combined AgNPs and Bepridil, which perturbed Ca^2+^ homeostasis in CLL cells [25]. Figure 4C (left) showed that the HG-3 cell viability was reduced to 51.59 ± 19.58% by Bepridil alone, to 30.25 ± 23.59% by AgNPs, and to 7.32 ± 10.64% by the combination as compared to controls set to 100%. The combination of AgNPs with Bepridil had synergistic results, as assessed by the Chou–Talalay method (Figure 4C, right top).

Further, we used the Compusyn program to calculate the dose reduction index (DRI), estimating the extent to which the dose of each agent used in the combination could be reduced to achieve a synergistic effect (CI < 1). We found that the DRI 50 values, which represent the magnitude of dose reduction obtained for the 50% growth inhibitory effect in combination, as compared to each drug, were (i) 3.5 and 3.2 for AgNPs and Venetoclax, respectively (Figure 4A, right bottom); (ii) 2.3 and 2.1 for AgNPs and Ibrutinib, respectively (Figure 4B, right bottom); and (iii) 9.3 and 9.0 for AgNPs and Bepridil, respectively (Figure 4C, right bottom).

### 3.6. AgNPs Conjugated with Rituximab Displayed Targeting Capability and In Vivo Anti-Leukemic Activity 

The greatest limitation of nanotechnologies’ applications in clinical translation is due to the non-specific distribution in vivo [26]. In order to develop a targeted therapy mediated by ligand-receptor specific affinity, we coated AgNPs with the anti-CD20 antibody Rituximab (AgNPs@Rituximab) and tested their anti-leukemic activity with the An V/PI assay against HG-3 cells after 24 h treatment in vitro.

**Figure 5:**
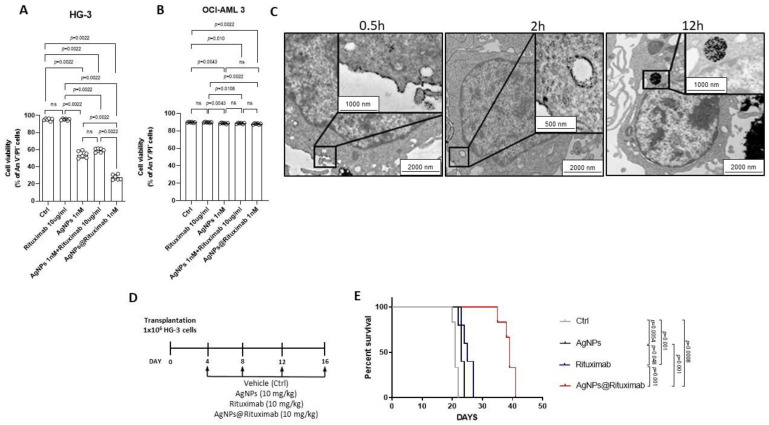
AgNPs conjugated with Rituximab display capability of targeting HG-3 cells and in vivo anti-leukemic activity. Column bar graph with data points of viable An V^−^/PI^−^ HG-3 cells (**A**) and OCI-AML 3 (**B**) treated for 24 h with vehicle control (Ctrl), Rituximab alone, AgNPs alone, AgNPs and Rituximab administrated simultaneously and separately, and AgNPs@Rituximab. Data are presented as the mean ± SD of six independent experiments. *p* values were calculated according to Mann–Whitney unpaired test. (**C**) Representative TEM images of ultrathin sections of HG-3 cells treated with AgNPs@Rituximab show particle uptake after 0.5 h (**left**), 2 h (**middle**), and 12 h (**right**) treatment. Magnified view of the selected regions inside cells is shown in the black boxes. (**D**) A schematic outline of the treatment schedule of NSG mice transplanted with HG-3 cells is shown. (**E**) Kaplan–Meier survival analysis in CLL xenograft models treated with vehicle control (Ctrl), AgNPs, Rituximab and AgNPs@Rituximab (*n* = 5). *p* values were calculated according to Mantel–Cox test.

The results in Figure 5A showed that AgNPs alone reduced cell viability to 54.42 ± 4.07% compared to 95.33 ± 1.41% of the control, whereas Rituximab alone did not affect cell viability (95.37 ± 0.97%). When unconjugated AgNPs and Rituximab were administered simultaneously, we observed a reduction in the cell viability similar to that induced by AgNPs alone. Notably, when cells were treated with AgNPs@Rituximab, cell viability was drastically lowered to 27.88 ± 3.07%, indicating that the conjugation with Rituximab potentiated the anti-leukemic effect of AgNPs (Figure 5A). To exclude non-specific effects of AgNPs@Rituximab on HG-3 cell viability, we tested AgNPs conjugated with an IgG1 antibody (AgNPs@IgG1) used as an isotype control. Results in Figure S4 showed that AgNPs@IgG1 did not affect HG-3 cell viability compared to AgNPs alone (56.32 ± 3.41% vs. 54.30 ± 3.57%). To demonstrate that AgNPs@Rituximab selectively targets CLL cells, we performed experiments using CD20-negative leukemic cells. We tested the effect of AgNPs@Rituximab for 24 h on the cell viability of the OCI-AML3 acute myeloid leukemia (AML) cell line. Results in Figure 5B showed that 10 μg/mL Rituximab alone did not have any effect on the OCI-AML3 cell viability compared with controls (89.80 ± 0.44% vs. 89.83 ± 0.43%). Unconjugated 1 nM AgNPs induced only a very modest reduction in OCI-AML3 cell viability, both when administered as a single agent (88.77 ± 0.61%) or in combination with Rituximab (88.68 ± 0.66%). A similar modest effect was also induced by AgNPs@Rituximab (88.03 ± 0.76%).

With the aim of demonstrating the advantages obtained from the conjugation of AgNPs with Rituximab, we performed a time-course analysis of TEM images to evaluate the targeting capability of AgNPs@Rituximab towards HG-3 cells compared with unconjugated AgNPs. As shown in Figure 5C, we demonstrated the adhesion of AgNPs@Rituximab on the HG-3 cell membrane after 0.5 h treatment. In addition, we observed a tendency of cell membrane invagination that documented the first step towards the internalization of AgNPs@Rituximab. Conversely, unconjugated AgNPs were not detectable in proximity to the HG-3 cell membrane (Figure S5). After 2 h treatment, the cell membrane was markedly coated with AgNPs@Rituximab, which was also found in the cytoplasm, suggesting their cellular uptake through the cell membrane, probably within endocytic-like structures. AgNPs@Rituximab appeared to be localized in the cellular matrix after 12 h exposure in close proximity to the nucleus (Figure 5C). After 2 h treatment, unconjugated AgNPs were found to be in contact with the mitochondrial structure, as also shown in Figure 3D, and at 12 h they appeared to be localized in the cellular matrix (Figure S5). These data documented the success of AgNPs@Rituximab multicomplex manufacturing and their better specificity compared to unconjugated AgNPs in targeting CLL cells.

Finally, with the aim of evaluating the efficacy of AgNPs@Rituximab in vivo, we performed experiments on xenograft models of CLL. Mice were divided into four groups and treated at days 4, 8, 12, and 16 after transplantation (day 0) with vehicle, AgNPs, Rituximab, and AgNPs@Rituximab (Figure 5D). The AgNPs@Rituximab treatment significantly improved the survival of transplanted mice compared to Rituximab and vehicle, with a median survival of 38, 27, and 21 days, respectively. Conversely, the administration of AgNPs alone had only a modest effect on median survival as compared to the control (23 days vs. 21 days), suggesting that conjugation with Rituximab is important for increasing AgNPs’ specificity towards leukemic cells in vivo (Figure 5E).

## 4. Discussion 

In the last few years, nanomedicine has been providing new concepts and approaches with potential therapeutic benefits in cancer. Still, we are in the emerging era of nanotechnology, and there is limited evidence for the use of nanotools in CLL therapy [5,6]. In the present study, we developed AgNPs with anti-leukemic activity in order to provide new insight into this topic and the basis for novel potential therapeutic avenues for CLL.

Our data revealed a strong susceptibility of primary CLL cells and the HG-3 CLL cell line to AgNPs cytotoxicity. The evidence that AgNPs significantly reduced CLL cell viability at lower concentrations compared to those used in solid tumor cell lines [27,28] suggested an improved tolerability profile of AgNPs for a potential translation in CLL therapy. Recent evidence identified mitochondrial integrity as the main target of AgNPs in several cancers [27,29]. In keeping with these observations, we found that AgNPs led to a significant imbalance of the Bax/Bcl-2 ratio in favor of the pro-apoptotic Bax molecule in HG-3 cells, indicating CLL cell susceptibility toward an intrinsic apoptosis pathway after AgNPs treatment. Reduced mitochondrial membrane potential and the increased activation of caspase-9 further suggested the induction of a mitochondrial-driven apoptosis caused by AgNPs in CLL cells. 

Mitochondrial apoptosis is a well-characterized mechanism triggered by various cellular signals, especially intracellular Ca^2+^ [30]. In CLL, Ca^2+^ signaling displays a controversial role since deregulated Ca^2+^ homeostasis represents both an important driver of cancer onset and progression, and a mediator influencing responses to therapies [31,32]. In this work, we found that CLL primary cells showed altered mRNA expressions of various Ca^2+^ channels as compared to normal B cells, suggesting a susceptibility of these leukemic cells to Ca^2+^-targeting agents. Ca^2+^ homeostasis has been identified as a target of AgNPs in other tumor types. In line with this observation, we showed that AgNPs induced the up-regulation of mRNA levels of Ca^2+^ channels localized on HG-3 cytoplasmatic [17] and mitochondrial membranes [18,19], as well as an increased intracellular Ca^2+^ levels due to an influx from the extracellular environment. 

Additionally, Ca^2+^ is closely involved in a bidirectional relationship with ROS generation [33]. Several studies have showed the capability of AgNPs to induce aberrant ROS production, resulting in the induction of the mitochondrial apoptosis pathway, in different cancer cell lines including chronic myeloid leukemia and breast, gastric, and pancreatic cancer [9,28,34,35,36]. We showed that AgNPs induced ROS overproduction in HG-3 cells after 2 h treatment, which was the time at which cell viability was already strongly reduced. These data suggested that an early Ca^2+^ perturbation followed by ROS overproduction might be responsible for CLL cell death induced by AgNPs. Similar to Ca^2+^ signaling, ROS production may drastically influence the tumoral cell fate. It is known that ROS, when they are generated at low levels, sustain cell viability, but when they are aberrantly produced they lead to cell death [37]. In CLL, the enhanced basal ROS levels and oxidative stress have been identified as a therapeutic vulnerability exploitable for target therapy [38]. This observation could explain why low concentrations of AgNPs are required to increase ROS levels beyond a sustainable threshold, thereby leading to mitochondrial apoptosis in CLL cells. The effects of AgNPs on CLL mitochondria were further evidenced by TEM analysis, which showed AgNPs in close proximity to mitochondria at the same time as the maximum ROS accumulation. Similarly, Bressan et al. showed that, in fibroblast cells, AgNPs are attracted near to the outer membrane of mitochondria after cellular uptake [39]. Overall, our data suggested that ROS production and cell death induced by AgNPs depended on different combined mechanisms: (i) physical interaction between AgNPs and mitochondrial structures and (ii) the perturbation of Ca^2+^ influx. However, further studies will be necessary to better characterize the interplay among mitochondrial apoptosis, Ca^2+^ perturbation, and ROS generation induced by AgNPs in CLL cells. These studies could reveal new targets for AgNPs with a therapeutic potential in CLL.

The Bcl-2 inhibitor Venetoclax and the BTK inhibitor Ibrutinib represent the main target agents used in CLL therapy able to provide durable responses in a single drug regimen. Despite this, some patterns of resistance have been described, prompting the development of combination strategies [40,41]. Here, we demonstrated the synergistic action of AgNPs and Venetoclax in reducing CLL cell viability and suggested that this effect is imputable to the combined action of AgNPs and Venetoclax in strengthening mitochondrial membrane damage, thus increasing MOMP-associated cell death mechanisms. Moreover, the combination of AgNPs and Ibrutinib resulted in a significant reduction in CLL viability compared to each single treatment. The rationale for the combination of AgNPs and Ibrutinib was the increased ROS production in CLL cells of Ibrutinib-treated patients [42], as well as the ability of Ibrutinib to sensitize AML cells to ROS inducer agents, synergizing with ROS-based first-line therapy Daunorubicin [24]. We found synergistic activity in the AgNPs/Ibrutinib treated cells, suggesting that AgNPs could potentiate the ROS-mediated cytotoxicity of Ibrutinib in HG-3 cells. We also examined the combination of AgNPs with the Ca^2+^ channel modulator Bepridil, which was shown to alter intracellular Ca^2+^ levels in CLL cells and was proposed as an attractive compound for the clinical development in CLL [25]. The AgNPs and Bepridil combination drastically reduced CLL cell viability when compared with the single drug treatment, probably due to their combined effects on Ca^2+^ homeostasis perturbation. Further studies evaluating the in vivo anti-leukemic effects of the combinations of AgNPs with Venetoclax, Ibrutinib, or Bepridil in mouse CLL models will be essential to provide a more solid rationale for the potential applications of AgNPs as new tools to improve targeted therapy in CLL. 

The major barrier for AgNPs clinical translation is the non-specific distribution with consequent off-target cytotoxic effects [7]. The key for enhancing the specific interaction of AgNPs with target cells is their conjugation with antibodies directed to molecules expressed on the cell surface of target cells [43,44]. In this study, we generated AgNPs conjugated with Rituximab, an anti-CD20 antibody used in CLL therapy [45,46,47]. Here, we demonstrated that HG-3 cells were markedly coated with AgNPs@Rituximab conjugates, which then appeared internalized within cells, indicating their specificity in targeting CLL cells and their improved cellular uptake. The high selectivity of AgNPs coated with anti-CD20 antibodies was also provided by Zhou et al., who demonstrated that these nanostructures were internalized more efficiently by CD20^+^ lymphoma cells than by CD20^−^ negative cells [48]. Strikingly, we showed that the anti-leukemic activity of AgNPs@Rituximab in vitro was strongly increased as compared to that induced by treatment with a single agent or with both unconjugated agents added simultaneously. These data suggested that Rituximab could function as a specific carrier of the anti-leukemic AgNPs, thus representing the rationale for the development of new potential CLL-targeted therapies. We demonstrated that AgNPs@Rituximab treatment is also effective in an in vivo CLL context. Specifically, survival rates of CLL xenograft models treated with AgNPs@Rituximab were significantly increased as compared to mice treated with the vehicle or unconjugated single agents. These data provided significant evidence that the conjugation of AgNPs with Rituximab could overcome the non-specific distribution of AgNPs in vivo by improving their selective internalization within leukemic cells and by allowing them to carry out anti-leukemic effects. The specificity proved by AgNPs@Rituximab towards leukemic B cells could also be relevant for reducing the cytotoxic side effects of AgNPs. 

## 5. Conclusions

In conclusion, the present study provided the first preclinical evidence of (i) the apoptotic effect of AgNPs and AgNPs conjugated with Rituximab on CLL cells in vitro; (ii) the ability of AgNPs to potentiate the cytotoxicity of Venetoclax, Ibrutinib, and Bepridil against CLL cells in vitro; and (iii) the improved efficacy, specificity, and bioavailability of AgNPs conjugated with Rituximab on in vivo CLL models.

These findings provide a framework for future studies aiming to assess nanotools for the therapeutic targeting of CLL. 

## Data Availability

All data and information concerning this study will be made available from the corresponding authors upon reasonable request.

## Supplementary Materials

The following supporting information can be downloaded at: https://www.mdpi.com/article/10.3390/cancers15143618/s1, Figure S1: UV-Vis and TEM characterization of AgNPs species; Figure S2: AgNPs induce apoptosis in CLL cells; Figure S3: AgNPs induce intrinsic apoptotic pathway in primary CLL cells; Figure S4: AgNPs induce intrinsic apoptotic pathway in primary CLL cells; Figure S5: Unconjugated AgNPs cellular uptake; Figure S6: Uncropped Western Blots; Table S1: CLL patients characteristics; Table S2: List of antibodies; Table S3: List of primers used for qRT-PCR.
