# Supplementary material for: Therapeutic Targeting Potential of Novel Silver Nanoparticles Coated with Anti-CD20 Antibody against Chronic Lymphocytic Leukemia
Source: Cancers (Basel). 2023 Jul 14;15(14):3618. doi: 10.3390/cancers15143618 (PMC10377400; doi:10.3390/cancers15143618)
Supplement: Supplementary file 1 [file cancers-15-03618-s001.zip › cancers-2444907-supplementary.pdf]

Supplementary Information

Therapeutic targeting potential of novel silver nanoparticles coated with anti-CD20 antibody against chronic lymphocytic leukemia

Adamo et al.

Contents:

- **Supplementary Figures**
1. *Figure S1.* UV-Vis and TEM characterization of AgNPs species
2. *Figure S2.* AgNPs induce apoptosis in CLL cells
3. *Figure S3.* AgNPs induce intrinsic apoptotic pathway in primary CLL cells
4. *Figure S4.* AgNPs induce intrinsic apoptotic pathway in primary CLL cells
5. *Figure S5.* Unconjugated AgNPs cellular uptake
6. *Figure S6.* Uncropped Western Blots

- **Supplementary Tables**
7. *Table S1.* CLL patients characteristics
8. *Table S2.* List of antibodies
9. *Table S3.* List of primers used for qRT-PCR.

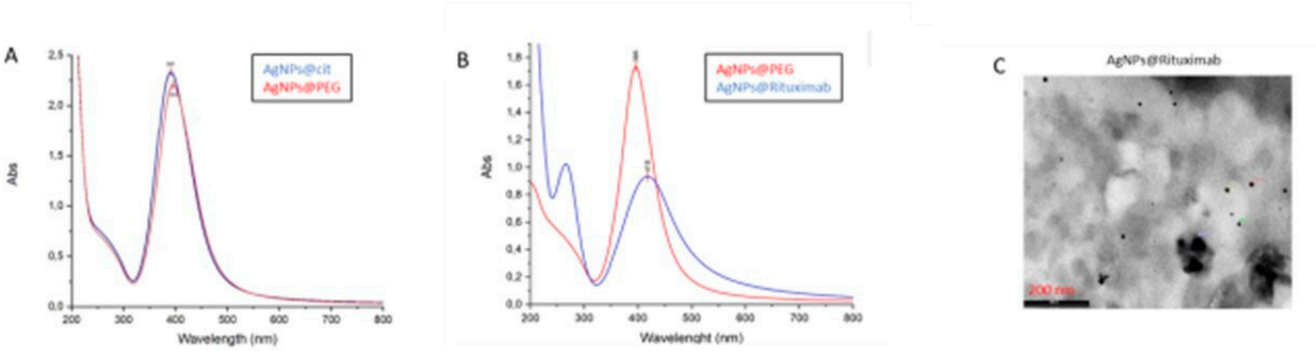

Figure S1. UV-Vis and TEM characterization of AgNPs species. A) The UV-Vis spectra of AgNPs citrate (blue) and after PEG conjugation (red). B) UV-Vis spectra of AgNPs PEG (in red) and after Rituximab conjugation (in blue). Note in both cases the red shift during the synthesis. C) TEM imaging analysis of AgNPs@Rituximab obtained with Phylips 208.

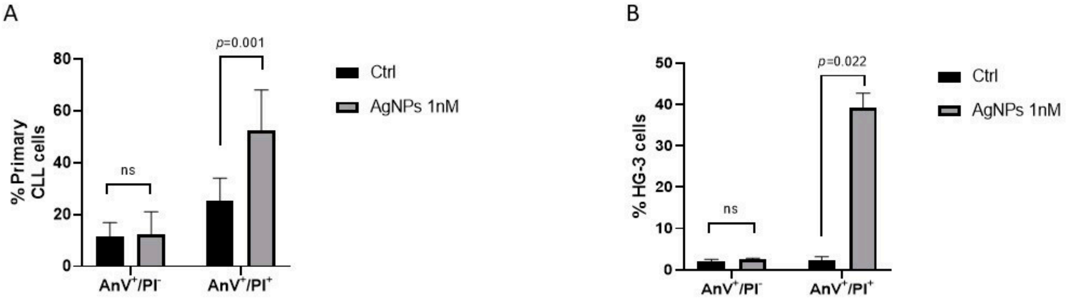

Supplementary Figure S2. AgNPs induce apoptosis in CLL cells. Percentage of AnV^+/PI^- (early apoptotic) and AnV^+/PI^+ (late apoptotic) primary CLL cells (A; N=18) and HG-3 cells (B; N=6) treated with vehicle control (Ctrl) and 1nM AgNPs for 24h. Data are presented as the mean ± SD. *P* values were calculated according to Wilcoxon paired test.

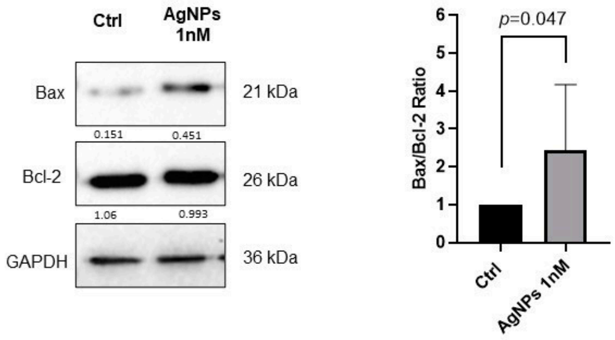

Supplementary Figure S3. AgNPs induce intrinsic apoptotic pathway in primary CLL cells. Left, representative Western blot of Bax and Bcl-2 in primary CLL cells (n=5) treated with vehicle control (Ctrl) and 1nM AgNPs for 24h . The number under each lane indicates the densitometry value of the band relative to GAPDH levels. Right, histogram of Bax/Bcl-2 ratio. Data are presented as the mean ± SD. *P* values were calculated according to Student t-test.

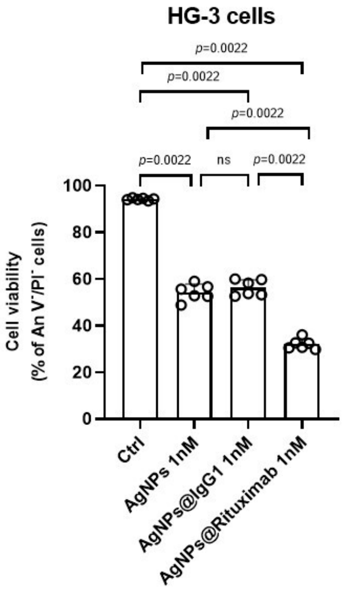

Supplementary Figure S4. AgNPs conjugated with Rituximab display capability of targeting HG-3 cells. Column bar graph with data points of viable An V^-/PI^- HG-3 cells treated for 24h with vehicle control (Ctrl), AgNPs alone, AgNPs@IgG1 and AgNPs@Rituximab. Data are presented as the mean $\pm$ SD of six independent experiments. *P* values were calculated according to Mann-Whitney.

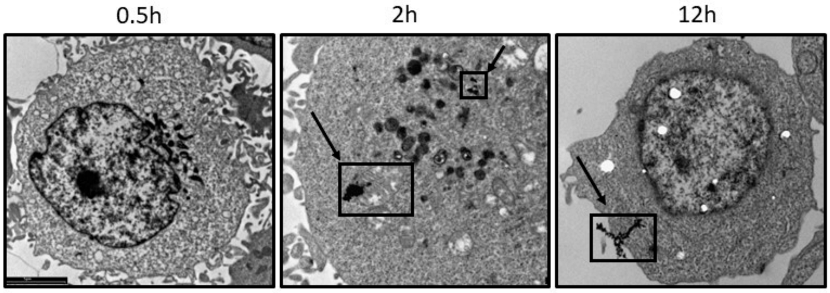

Supplementary Figure S5. Unconjugated AgNPs cellular uptake. Representative TEM images of ultrathin sections of HG-3 cells treated with 1nM AgNPs for 0.5h, 2h and 12h. Particles are visible in the cells as black and electron-dense spots indicated by arrows.

Uncropped blots for Figure 1D

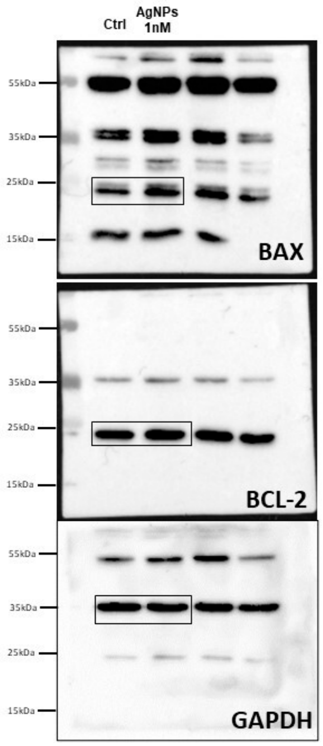

Uncropped blots for Figure 1F

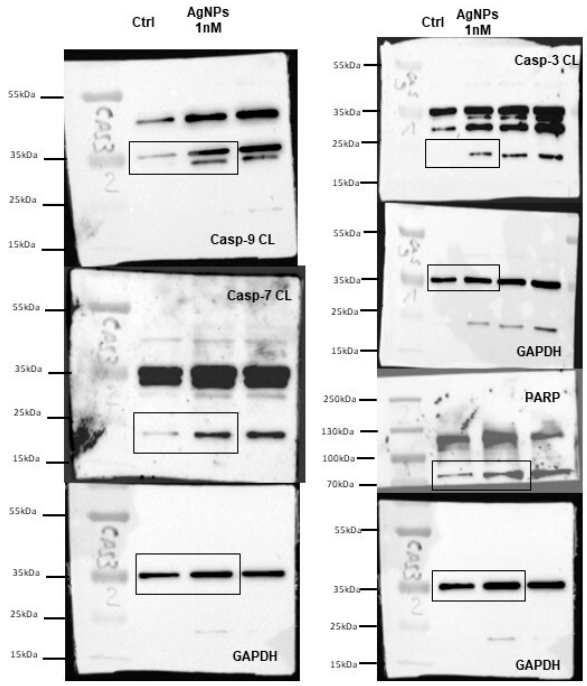

Uncropped Blots for Figure S3

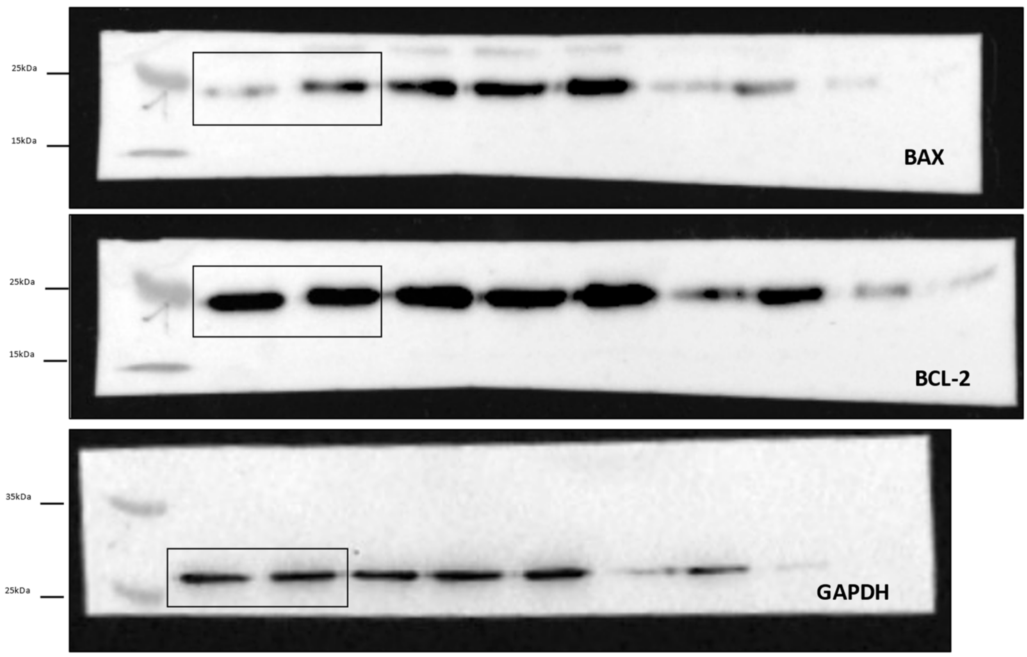

Figure S6. Uncropped Western Blots.

Supplementary Table S1. CLL patients characteristics

Pt. No#	Age	Sex	Rai/Binet	FISH	IGHV	NOTCH1	P53	SF3B1	MYD 88
001	61	M	I/B	DEL 13	UM	M	UM	UM	UM
002	58	M	II/B	NORM	UM	M	UM	UM	UM
003	49	M	II/B	DEL 13	UM	M	UM	UM	ND
004	77	M	0/A	DEL 11; TRIS 12	UM	M	UM	UM	UM
005	65	F	II/B	DEL13	M	UM	ND	UM	UM
006	61	M	0/A	DEL 13	UM	UM	UM	UM	UM
007	67	M	0/A	ND	M	UM	ND	UM	UM
008	83	M	0/A	TRIS 12; DEL 14	UM	M	UM	UM	UM
009	75	M	0/A	NORM	UM	M	UM	UM	ND
010	87	F	I/A	NORM	UM	UM	ND	UM	UM
011	70	F	0/A	ND	UM	UM	ND	UM	UM
012	62	M	0/A	DEL 13	M	UM	UM	UM	ND
013	67	F	0/A	DEL 13	M	UM	ND	ND	ND
014	71	M	0/A	DEL 13	UM	UM	ND	UM	UM
015	51	F	II/A	NORM	M	M	ND	UM	UM
016	69	M	0/A	DEL 13	M	UM	UM	ND	ND
017	54	M	II/B	NORM	M	M	UM	UM	ND
018	64	F	I/B	NORM	M	M	ND	UM	UM
019	77	F	0/A	DEL 13	UM	M	ND	UM	UM
020	84	F	0/A	DEL 11, DEL 13	UM	UM	ND	UM	UM
021	80	F	II/B	NORM	M	UM	UM	ND	ND
022	68	M	II/B	NORM	M	UM	ND	UM	ND
023	86	M	III/C	DEL 11	UM	UM	UM	ND	ND

Supplementary Table S2. List of antibodies

Target protein	Clone	Target Species	Supplier	Catalog number	Application
CD45	J33	Human	Beckman Coulter	B36294	Flow cytometry
CD5	BL1a	Human	Beckman Coulter	A21690	Flow cytometry
CD19	J3-119	Human	Beckman Coulter	A07770	Flow cytometry
Caspase-9	Polyclonal	Human	CST	#9502	WB
Caspase-3	Polyclonal	Human	CST	#9662	WB
Caspase-7	Polyclonal	Human	CST	#9492	WB
PARP	Polyclonal	Human	CST	#9542	WB
BCL-2	124	Human	Dako	M0887	WB
BAX	E-5	Human/Mouse	SCB	sc-166410	WB
GAPDH	GAPDH-71.1	Human/Mouse	Sigma	G8795	WB

Abbreviations: CTS, Cell Signaling Technology; SCB, Santa Cruz BioTechnologies; WB, western blot

Supplementary Table S3. List of primers used for qRT-PCR.

Target gene	Forward primer (5' to 3')	Reverse primer (5' to 3')
KCNN4	CAT TCC TGA CCA TCG GCT ATG G	GCC TTG TTA AAC TCC AGC TTC CG
MCU	AGC CTA TCT CTG ACT CTG TTG G	GTG GTC GTA CGT GGT ATG TT
VDAC1	ACT GCA AAA TCC CGA GT	AGC GCG TGT TAC TGT TTC CT
IP3R3	ACT TCC TGC ACA CCG TCA TT	GCC TTC ATC ATG TCC AGC AG
ATP2A2	GGA CTT TGA AGG CGT GGA TTG TG	CTC AGC AAG GAC TGG TTT TCG G
